# Evaluation of a Novel Psychological Intervention Tailored for Patients With Early Cognitive Impairment (PIPCI): Study Protocol of a Randomized Controlled Trial

**DOI:** 10.3389/fpsyg.2020.600841

**Published:** 2020-12-23

**Authors:** Urban Ekman, Mike K. Kemani, John Wallert, Rikard K. Wicksell, Linda Holmström, Tiia Ngandu, Anna Rennie, Ulrika Akenine, Eric Westman, Miia Kivipelto

**Affiliations:** ^1^Center for Alzheimer Research, Division of Clinical Geriatrics, Department of Neurobiology, Care Sciences, and Society, Karolinska Institutet, Stockholm, Sweden; ^2^Medical Unit Medical Psychology, Allied Health Professionals, Karolinska University Hospital, Stockholm, Sweden; ^3^Medical Unit Ageing, Allied Health Professionals Function, Karolinska University Hospital, Stockholm, Sweden; ^4^Stress Research Institute, Department of Psychology, Stockholm University, Stockholm, Sweden; ^5^Department of Clinical Neuroscience, Karolinska Institutet, Stockholm, Sweden; ^6^Public Health Promotion Unit, Finnish Institute for Health and Welfare, Helsinki, Finland; ^7^Theme Aging, Karolinska University Hospital, Huddinge, Sweden; ^8^Department of Neuroimaging, Centre for Neuroimaging Sciences, Institute of Psychiatry, Psychology and Neuroscience, King’s College London, London, United Kingdom; ^9^Institute of Public Health and Clinical Nutrition, University of Eastern Finland, Kuopio, Finland; ^10^Ageing and Epidemiology (AGE) Research Unit, School of Public Health, Imperial College London, London, United Kingdom

**Keywords:** psychological intervention, prevention, cognitive impairment, randomized controlled trial (RCT), cognitive behavioral therapy (CBT), acceptance commitment therapy (ACT)

## Abstract

**Background:**

Individuals with early phase cognitive impairment are frequently affected by existential distress, social avoidance and associated health issues (including symptoms of stress, anxiety, and depression). The demand for efficient psychological support is crucial from both an individual and a societal perspective. We have developed a novel psychological intervention (Psychological Intervention tailored for Patients with Cognitive Impairment, PIPCI) manual for providing a non-medical path to enhanced psychological health in the cognitively impaired population. The current article provides specific information on the randomized controlled trial (RCT)-design and methods. The main hypothesis is that participants receiving PIPCI will increase their psychological flexibility (the ability to notice and accept interfering thoughts, emotions, and bodily sensations without acting on them, when this serves action in line with personal values) compared to participants in the active control (cognitive training) group and the waiting list control group. The secondary hypotheses are that participants receiving PIPCI will improve psychological health (stress measures, quality of life, depression, and general health) compared to participants in the active control group and the waiting list control group.

**Materials and Methods:**

This three-arm RCT will recruit participants from the cognitive centers at Karolinska University Hospital in Stockholm and randomize approximately 120 individuals in the early phase of cognitive impairment to either an experimental group (psychological intervention once a week for 10 weeks), an active control group (cognitive training once a week for 10 weeks) or a waiting list control group. Intervention outcome will be evaluated with self-report questionnaires on physical and psychological aspects of health, cognitive assessment, biological markers (obtained from blood and saliva) and health care costs. Assessments will be performed at pre- (1 week before the interventions) and post-intervention (1 week after the interventions), as well as at a 6-month follow-up.

**Discussion:**

The development of a potentially feasible and effective psychological intervention tailored for early phase cognitive impairment (PIPCI) has the potential to advance the non-pharmacological intervention field. This is especially important given the extensive burden for many affected individuals and their families and the current lack of effective treatments. If the psychological intervention discussed here shows feasibility and efficacy, there is potential for far-reaching healthcare implications for patients with early cognitive impairment at risk of developing dementia.

**Clinical Trial Registration:**

ClinicalTrials.gov: NCT04356924. Date of registration: April 22, 2020. URL: https://clinicaltrials.gov/ct2/show/NCT04356924.

## Introduction

The burden of cognitive impairment (CI) is substantial globally. CI often hinders affected people to act independently in daily life, and the close family members often carry a large burden. A considerable societal challenge is to promote the maintenance of cognitive health in order to prevent cognitive disability in the aging population (Wallin et al., 2018). Individuals diagnosed with subjective cognitive decline (SCD, with unimpaired performance on cognitive tests) (Jessen et al., 2014), mild CI (MCI, impaired performance on cognitive tests) (Winblad et al., 2004), prodromal dementia or dementia are frequently affected by existential distress and associated health issues (such as symptoms of stress, anxiety, and depression), as well as social stigma and avoidance. The lack of efficient disease-modifying drugs has increased the interest in dementia prevention and the promotion of psychological health among those at risk/early stages of dementia. Thus, efficient psycho-social interventions are needed both from an individual- as well as a societal perspective (Wallin et al., 2018).

Adaptation to changing life circumstances is important to individuals with CI to maintain functioning and good health (Chatterji et al., 2015). Importantly, modifiable risk factors may account for approximately 35% of dementia cases (Livingston et al., 2017), and beneficial lifestyle changes hold the potential to substantially reduce the number of expected cases (Norton et al., 2014). The Finnish Geriatric Intervention Study to Prevent Cognitive Impairment and Disability (FINGER) study was the first multidomain lifestyle intervention that showed positive effects on cognitive functioning among older adults at risk of dementia (Ngandu et al., 2015). FINGER is now globally used as a prevention model, and more than 30 countries have joined the World-Wide FINGERs network. However, it is well known that adherence influences intervention efficacy, and unfortunately, adherence to lifestyle recommendations is often lower than to medication (Coley et al., 2019). Health behaviors are also generally difficult to change, and several common characteristics, including poorer cognition and depressive symptoms, may result in lower adherence to multidomain lifestyle interventions among older at-risk individuals (Coley et al., 2019). In addition, depression or depressive symptoms are common in clinical settings, potentially reaching a prevalence of over 40% for individuals with MCI, and sub-clinical depressive symptoms are associated with the increased use of healthcare (Meeks et al., 2011).

Although the demand for psychological support is significant in the cognitively impaired population, there is a lack of efficient psychological interventions to maintain and improve psychological health and to facilitate adherence to health-promoting behavioral change (Wallin et al., 2018). Cognitive training for individuals with MCI has been evaluated in a randomized controlled trial (RCT) and compared with a group intervention targeting psycho-social support using cognitive behavioral therapy (CBT) techniques (Belleville et al., 2018). Results showed that cognitive training improved the use of strategies in daily life, but neither cognitive training nor CBT improved patient mood or well-being. In addition, a systematic review stated that modified CBT approaches have shown promise in improving the quality of life in individuals with MCI and early dementia (Regan and Varanelli, 2013). However, although previous research has shown some promise, the authors call for studies with a more rigorous methodology, and larger RCT studies with better control group conditions. Thus, previous research on psychological treatments for individuals with early onset of CI with strong methodology is still limited. Increased knowledge of how to prevent, cope, and intervene with CI is therefore utterly needed, especially for those that struggle with adherence to lifestyle changes (Wallin et al., 2018).

To enhance health and functioning among individuals in an early phase of CI, we have developed a new psychological intervention. The objective of this intervention approach is to (a) improve psychological flexibility, or the ability to behave in accordance with personal values and long-term goals also in the presence of negative experiences (Wicksell et al., 2010), and (b) increase the patient’s motivation for meaningful life-style changes as defined in the FINGER concept, and for them to live their life in correspondence with personal goals. The current intervention manual is developed within the CBT tradition and takes into account the known challenges for cognitively affected individuals, and therefore for example includes additional sessions and the systematic utilization of reminders, validation techniques, repetition, and concrete examples. CBT represents a wide variety of interventions aimed at decreasing distress as well as increasing emotional, psychological, physical and social functioning (Turk et al., 1983). Recent developments within CBT, particularly acceptance and commitment therapy (ACT), emphasize the utility of acceptance and mindfulness strategies in order to facilitate value-based action when facing subjective difficulties in life that are beyond direct control, which in part stands in contrast to a number of interventions that focus on reduction or control of symptoms (Hayes et al., 2006). ACT has been empirically evaluated for a variety of psychiatric/somatic conditions, such as chronic pain and depression (Hayes, 2016). In brief, the treatment objective in ACT is to improve psychological (or behavioral) flexibility, defined as the ability to notice and accept interfering thoughts, emotions and bodily sensations without acting on them, to facilitate behavior in accordance with personal values and long-term goals also in the presence of those negative experiences (Wicksell et al., 2010). Furthermore, psychological flexibility is a key component in the change process in ACT treatment, mediating the treatment effect on pain interference, catastrophizing and anxiety (Wicksell et al., 2011; Kemani et al., 2016). ACT has also been RCT-evaluated for chronic pain by members of the current research group (Wicksell et al., 2008), and the most recent evaluation of ACT by the American Psychological Association (Division 12) was strongly supportive of ACT as treatment for “chronic or persistent pain in general” (American Psychological Association, 2010). Notably, despite the state of evidence, accessibility to CBT and ACT is low, particularly for individuals with CI such as SCD or MCI. In addition, these treatments are generally not adapted for individuals with CI. If the current intervention is effective, it has the potential to enhance psychological adjustment and health in individuals with CI. More specifically, the proposed intervention may improve the ability to adjust to the implications of cognitive decline, which potentially may also delay disease progression, and have significant health economic effects.

### Objectives and Outcomes

We will evaluate the efficacy of the psychological intervention (PIPCI) manual in an RCT comparing the intervention with both an active control group condition (cognitive training), and a waiting list control group condition. The primary objective is to evaluate intervention-related changes in psychological flexibility measured with the second version of the Acceptance and Action Questionnaire (AAQ-II) (Bond et al., 2011). The secondary objectives are to further evaluate intervention-related changes with measures of psychological health, cognitive measures and biological markers (obtained from blood and saliva). The behavioral measures will also be evaluated in relation to changes in biological markers (obtained from blood and saliva; see section “Materials and Methods” for further information). We will also evaluate how the participants experience the intervention, and how they handle preventive actions, using qualitative methods.

### Hypotheses

#### Main Hypothesis

1. The main hypothesis states that participants receiving PIPCI will increase their psychological flexibility compared to participants in the active control group and the waiting list control group and that this effect will be maintained at 6 months follow-up.

#### Secondary Hypotheses

1. The secondary hypotheses state that participants receiving PIPCI will improve psychological health (stress measures, quality of life, depression, and general health), and increase their telomerase activity compared to participants in the active control group and the waiting list control group.
2. An additional secondary hypothesis state that participants receiving PIPCI, and participants in the active control group, will improve on the cognitive test measures compared to the waiting list group. However, we do not have any directed hypothesis comparing PIPCI and the active control group.

Exploratory research questions (without any directed hypotheses)

1. Can we identify significant pre-intervention predictors for intervention outcomes?
2. How do participants perceive the intervention?
3. Is PIPCI cost-effective and does it impact health-economic variables?

The current protocol article aims to provide a description of the conceptual model behind the novel psychological intervention manual as well as specific information on the study design, materials and methods.

## Materials and Methods

### General Design

The psychological intervention manual was developed with a developmental mixed-method according to the Medical Research Council guidance (Supplementary Material) (Moore et al., 2015). Thus, the developmental and innovative parts of the research project used an interactive design approach where both patients’ and expert clinician’s interactions are vital. The evaluation trials will be conducted according to the Standard Protocol Items: Recommendations for Interventional Trials (SPIRIT) guidelines (Chan et al., 2013) and the Consolidated Standards of Reporting Trials (CONSORT) statement (Schulz et al., 2010). The present study protocol is registered at ClinicalTrials.gov with identifier: NCT04356924 (URL: https://clinicaltrials.gov/ct2/show/NCT04356924).

### Patients

Patients will be recruited from the Cognitive Centers at the Karolinska University Hospital, Solna, and Huddinge within the Stockholm metropolitan area in Sweden, where they partake in cognitive examination [including neuropsychological assessments, anamnestic interviews, neurophysiological instrumental evaluations (such as MRI), and tests for biological markers (such as cerebrospinal fluid, CSF) etc.]. Eligible patients are individuals younger than 70 years that have been diagnosed with SCD or MCI through multidisciplinary consensus agreements according to the ICD-10 (World Health Organization, 1992), and in conjunction with consensus classification for MCI (Winblad et al., 2004). Patients that express interest in participating will be evaluated using a semi-structured interview (based on the Mini-International Neuropsychiatric Interview, MINI) (Sheehan et al., 1998), and those that fulfill the inclusion criteria are then randomized to one of the three intervention arms.

Criteria for inclusion

• <70 years.
• SCD or MCI diagnosis. All cognitive MCI-subtypes are eligible.
• Mild to moderate psychological symptoms that are indicated to be related to the patient’s CI. The psychological symptoms should affect the patients daily living and behavior, exemplified by avoidance behavior, social anxiety, and perceived stigmatization.
• Fluency in the Swedish language.
• The patients should have access to a mobile telephone to be able to receive reminders via Short Message Service (SMS).
• Signed informed consent.

Criteria’s for exclusion

• Dementia diagnosis and/or occurrence of serious illness and/or injury that requires immediate investigation or treatment of another type, or which is expected to worsen in the coming year (i.e., not including dementia).
• Participation in other psychological treatment over the past 6 months.
• Severe psychiatric comorbidity (e.g., high suicide risk), and/or severe psychiatric disorder. This will be assessed in the MINI evaluation and during the clinical cognitive examination).
• Anti-depressant medication introduced or alterations in dosage <6 months ago (i.e., un-stable dose).
• Mini Mental State Examination (MMSE) score <26 and/or a Montreal Cognitive Assessment (MoCA) score <24.
• Stroke or head trauma <6 months ago.
• Present substance abuse diagnosis.

### Evaluation Phase

#### Randomization

The fulfillment of the inclusion and exclusion criteria will be assessed through clinical evaluations at the diagnostic team (clinicians) round. All patients eligible for study inclusion will then receive verbal and written information about the project. In the next step, patients that express interest in the project sign an informed consent and will be evaluated in a semi-structured clinical interview, the Mini International Neuropsychiatric Interview (MINI), conducted by a psychologist at the Medical Unit, Medical Psychology at Karolinska University Hospital, Stockholm, Sweden. Patients that fulfill the inclusion criteria and are considered suitable for intervention are randomized. For the randomization process, we will engage the Karolinska Trial Alliance^1^, Stockholm, Sweden, an independent professional clinical research center at Karolinska University Hospital specialized in clinical trials. Blocking will be used with each block consisting of thirty participants, this is to ensure that the number of participants will be of approximately the same size. We will use two strata, and that will be on cognitive diagnosis (SCI and MCI) and gender.

#### RCT Design and Groups

Group 1 will receive the psychological intervention (experimental group), group 2 will partake in cognitive training (active control group), and group 3 will be randomized into a waiting list control group (Figure 2). Participants need to be present for at least 75% of the intervention and complete at least 75% of the homework to be considered adherent. All evaluation assessments will be conducted by blinded assessors to the extent possible. Participants cannot be blinded to group assignment, which is typical of non-pharmacological intervention trials. However, they will not be informed of the hypotheses or of which intervention (i.e., experimental intervention and active control group intervention) was considered the experimental or control condition, to minimize expectancy effects.

• Group 1, Experimental condition: Psychological intervention (PIPCI). The intervention is an adjusted (to the cognitively impaired individual) combination of CBT and ACT, including validation strategies, and psychoeducation. One focus is to increase the patient’s internal motivation for lifestyle changes, and for them to live their life in correspondence with personal goals (see section “Introduction”). Each session includes mindfulness exercises (5–10 min per occasion) that gradually become more advanced. The psychological intervention consists of 10 sessions (55 min per occasion), once a week, where the patient meets a psychologist face-to-face (either licensed or under training to be licensed, after having worked as a psychologist for 1 year under supervision) once a week. In between sessions, patients are expected to do homework assignments related to the contiguous sessions (2 × 45 min per week). Reminders will be sent to the patient’s mobile telephone via SMS. See Table 1 for the intervention structure and content overview.

**TABLE 1:** Description of the sessions in the psychological intervention.

Session	Topic	Intervention strategies
**Psychiatric evaluation and goal settings**		
Pre-intervention session	Psychiatric evaluation	Evaluation with a semi-structured interview based on MINI. After a maximum of three workdays, the patient will receive a decision of inclusion or exclusion. This part is conducted before the randomization.
1	Behavior analysis	The intervention rational is presented including a tentative functional analysis of concrete situations, cognitive processes, emotions, behaviors, strategies and short- and long-term consequences of these behaviors. Also, a first discussion is held on individual intervention goals.
**Phase 1: preparation for change**		
2	Introduction to intervention	Identification of patient specific discomfort (undesirable thoughts, feelings and bodily sensations) in different situations (antecedents [A]), and an introduction to present moment focus as a way to facilitate behavioral change.
3	Continuation of the ABC model	Continuation of session 2 with a greater focus on the behaviors (B) and consequences (C) related to the previously discussed antecedent (A) situations. The therapist focuses on emotional validation and in keeping the communication straightforward and concrete.
4	Psychoeducation	Presentation of a psychoeducative model on cognitive impairment, as well as discussions of stigma and how to more effectively handle potential guilt and shame. Also, helpful cognitive strategies are discussed with respect to the patient’s specific cognitive impairments.
**Phase 2: life values**		
5	Formulation of life values	The psychologist introduces life values as ongoing aspects of life that are highly valued, as qualities we want our lives to be about, that we want to fill our lives with, and as the type of persons we want to be. And, encourages formulation of such values in different life domains, while helping the patient to understand values as a broader concept, compared to goals.
**Phase 3: shift of perspective**		
6	The direction of life values	Introduction to strategies aimed to help the patient to deal with discomfort, while at the same time encouraging behavioral steps in a valued direction, e.g., taking steps to be more socially active.
7	Value-oriented behaviors	Further formulation and clarification of life values and introduction to the formulation of concrete value-oriented behaviors, i.e., short- and long-term goals in line with the personally formulated values.
**Phase 4: Behavioral activation, exposure, and strategies**		
8	Value-oriented behavioral activation	Exercises are presented aimed to encourage values consistent behaviors and to adopt an accepting attitude toward situational discomfort (e.g., negative thoughts and related emotions) that is not directly alterable.
9	Value-oriented behaviors and strategies	Repetition of acceptance strategies is done, to help the patient to establish situational conditions in everyday life that promote the ability to act effectively in line with life values, also in the presence of discomfort. Practice in using ACT-based problem-solving strategies is also covered.
**Phase 5: Closure**		
10	Summary and closure	A summary of the intervention is done, including reflections on closure and a discussion of questions that the patient may have. Also, a plan for relapse prevention is discussed and formulated, including the need for repetition and use of strategies to handle discomfort in the future.
• Group 2, active control condition: Computer-based training tasks administered with a difficulty level adapted to patient performance. The cognitive training method that will be used targets executive control (i.e., ability to coordinate thoughts and actions in accordance with internal goals) (Miyake et al., 2000). The tasks are intended to train specific types of executive functions: “Shifting” (flexibly switching between different stimuli), “Updating” (organizing information in working memory and actively exchanging old information with new) and “Inhibition” (actively inhibiting an automatic or more dominant response). An active control group is important to include to create a placebo-like condition in addition to the waiting list condition (Van De Ven et al., 2016). It is important that the participants believe that they will improve to facilitate motivation, attention, or other factors that will increase performance as well. In addition, the active control group will receive the same amount of sessions as the experimental group, thus the active control group also consists of 10 sessions (55 min per occasion), once a week. On those occasions, the patient will meet a psychologically trained (i.e., psychology student under clinical training or with an MSc in psychology) research assistant that coaches the patients during the cognitive training. Between sessions, patients are supposed to take two walks (45 min per occasion to meaningfully match the home exercises in the experimental group). Reminders will be sent to the patient’s mobile telephone via SMS.
• Group 3, waiting list condition: This group only receives regular health information that is provided after the extended cognitive examination at the Cognitive Center. After the finalization of the post-intervention evaluations, this group will be offered to participate in one of the active interventions by their preference. We will conduct additional post-intervention assessments also for those individuals in this group that accept this offer to increase the power of the intervention evaluation.

#### Standardization of Procedures

The intervention manuals and additional materials will be available for all participating psychologists in a web-based encrypted forum. Adherence to the intervention manual is monitored by several study features: 1. Only psychologists with previous therapeutic experience in CBT/ACT will interact with patients. 2. Psychologists undergo intensive training on the intervention manual ahead of the intervention phase. 3. Regular supervision by experienced psychologists/psychotherapists. 4. An adherence checklist in the intervention manual, where the psychologist takes structured notes after each session. 5. Via randomized auditory recordings of the sessions that are reviewed by two independent clinical psychologists.

#### RCT Assessments

**FIGURE 1:**
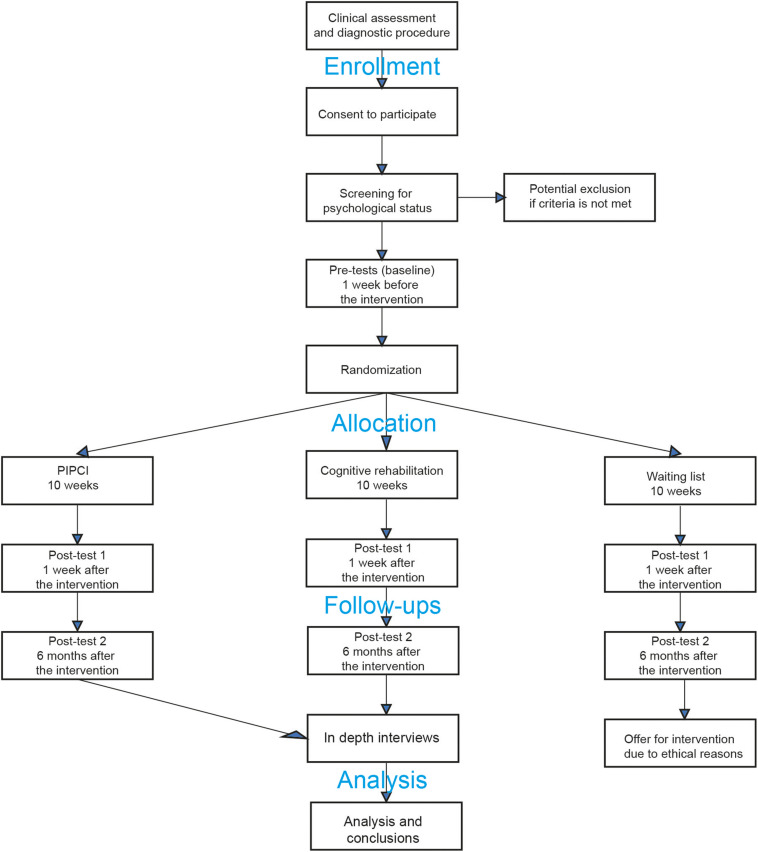
Illustrating a CONSORT (Schulz et al., 2010) inspired flow-chart of the trial design. PIPCI, Psychological Intervention tailored for Patients with early Cognitive Impairment.

Data will be collected at pre-intervention (1 week before the interventions), post-intervention (1 week after the interventions) and 6 months follow-up (Figure 1). Blinded independent psychology graduated (MSc) assistants, psychology students under clinical training, or licensed psychologists will carry out the evaluation assessments to avoid biased estimates of treatment effects. Outcome analysis will be adjusted for pre-intervention baseline. Clinical data from the patients’ extended cognitive examinations will be extracted from their patient journals and statistically modeled to predict intervention outcomes.

During pre-intervention assessments, data will be collected on demographics such as age, gender, education, employment status, and duration of cognitive complaints. In addition, the Childhood Trauma Questionnaire (CTQ) will be assessed to evaluate if early life trauma can predict treatment outcome (Bernstein et al., 2003).

During post-intervention assessments, data will be collected with the Client Satisfaction Questionnaire (CSQ) (Kwon et al., 2017) in order to evaluate the participant’s satisfaction with the interventions.

**FIGURE 2:**
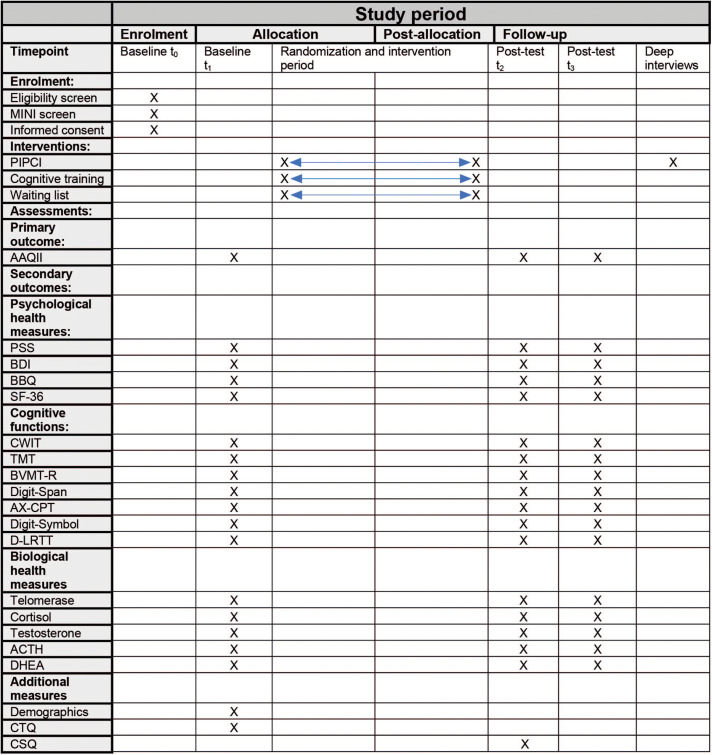
Illustrating a SPIRIT diagram of enrolment, intervention, and assessments. PIPCI, Psychological Intervention tailored for Patients with early Cognitive Impairment; MINI, Mini International Neuropsychiatric Interview; AAQ-II, Acceptance and action questionnaire – second version; PSS, Perceived Stress Scale; BDI, Becks Depression Inventory; BBQ, Brunnsvikens Brief Quality of Life Scale; SF-36, Short Form Health Survey; CWIT, Color Word Interference Test; TMT, Train Making Test; BVMT-R, Brief Visiospatial Memory Test Revised; AX-CPT, AX-Continuous Performance Task; D-LRTT, Deary-Liewald Reaction Time Task; ACTH, adrenocorticotropic hormone; DHEA, dehydroepiandrosterone; CTQ, Childhood Trauma Questionnaire; CSQ, Client Satisfaction Questionnaire.

As recommended by the SPIRIT statement, a schematic overview of the timeline, and evaluation components is shown in Figure 2.

### Outcome for the Pre- and Post-evaluations

#### Primary Outcome Measures

• Psychological health measures: Our primary outcome measure is psychological flexibility measured with the total score of the AAQ-II (Bond et al., 2011). This outcome is motivated by the fact that psychological flexibility is the essential target of our psychological intervention and has a strong history of evidence, as detailed in the introduction.

#### Secondary Outcome Measures

• Psychological health measures: Stress-related symptoms reported with the Perceived Stress Scale (PSS) (Lee, 2012), depressive symptoms with the Becks Depression Inventory (BDI) (Dozois et al., 1998), quality of life with the Brunnsviken Brief Quality of Life Scale (BBQ) (Lindner et al., 2016), and general health with the Short Form Health Survey (SF-36) (Ware, 2000).
• Cognitive functions: Executive functions with the Color-Word Interference Test (CWIT) and the Trail Making Test (TMT) from Delis-Kaplan Executive Function System (D-KEFS) (Delis et al., 2001), non-verbal episodic memory with the Brief Visuospatial Memory Test-Revised (BVMT-R) (Benedict et al., 1996), attention/working-memory with Digit-Span from the Wechsler Adult Intelligence Scale (WAIS-IV) (Wechsler, 2008), and the computer-based AX-Continuous Performance Task (CPT) (Braver and Cohen, 2000), processing speed with Digit-Symbol from WAIS-IV (Wechsler, 2008), and psychomotor speed with the Deary-Liewald Reaction Time Task (D-LRTT) (Deary et al., 2011).
• Biological health measures: Telomerase (the enzyme involved in maintaining telomere length, among other things) activity by modified real-time telomeric repeat amplification protocol (Hou et al., 2001). This outcome is motivated by previous research showing that biological measures, such as telomere length are related to aging and differences in such biological marker has been coupled with differences in the prevalence of psychiatric, cognitive, and somatic conditions (Han et al., 2019). Telomerase activity may also be associated with improvement to psychological treatment (Månsson et al., 2019). In addition, we will also evaluate saliva measures of cortisol, testosterone, adrenocorticotropic hormone (ACTH), and dehydroepiandrosterone (DHEA) (Kalman and Grahn, 2004).
• Costs-effectiveness: Health-economic impact is measured in Quality Adjusted Life Years (QALY’s) (Torrance and Feeny, 1989).

#### Reliability and Validity of Outcome Measurements

For AAQ-II the mean alpha coefficient for the items is 0.84 and the scale has shown acceptable test-retest reliability (0.79–0.81) (Bond et al., 2011). PSS has repeatedly shown alpha coefficients of >0.70 and the test-retest reliability has been assessed in four studies and met the criterion of >0.70 in all cases (Lee, 2012). BDI-II has shown high internal consistency with alpha coefficient >0.90, furthermore, it has shown adequate validity in terms of diagnostic discrimination as well as validity in terms of factorial and content validity (Dozois et al., 1998). BBQ has shown satisfactory reliability both in terms of inter-item correlation and Cronbach’s alpha, as well as regarding test-retest reliability in the short term. An intra-class correlation coefficient of 0.82, indicate high test-retest reliability (Lindner et al., 2016). The SF-36 is a well-validated and reliable scale that often reaches alpha coefficients of >0.80, and has been used in multiple settings and shown both discriminatory and predictive ability (Ware, 2000). For the cognitive measures, information regarding reliability and validity are referred to in the test manuals above. For the biological measures, the analytical methods are yet to be decided, but we aim to employ methods with adequate psychometric properties.

### Power Estimation and Sample Size

**FIGURE 3:**
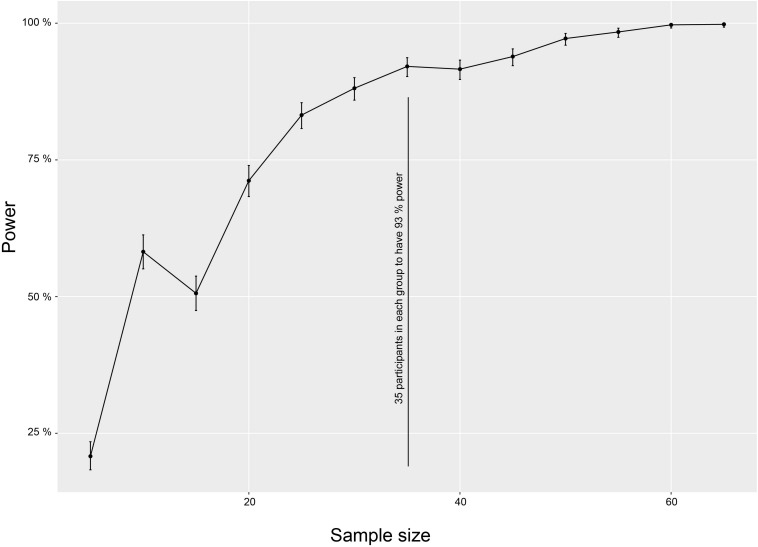
Power calculation. The sample size estimate is based on changes in our primary outcome, AAQ-II. The figure illustrates a power curve function to explore trade-offs between power and sample size. The analysis showed that a significant difference between the experimental group and the active control group require at least 35 participants (to have 93% power) in each group. Error bars = standard deviation.

No studies are available directly comparing CBT/ACT in a cognitively impaired population with an active control condition using AAQ-II. Therefore, we designed our RCT as a superiority trial with enough statistical power to detect a difference in outcome between treatments (if present) with a medium effect size. We chose this minimum difference because such a difference is important based on a patient’s perspective or clinical knowledge. Expecting larger differences in outcome does not seem realistic (Regan and Varanelli, 2013) and might result in an underpowered study while detecting smaller differences is of less relevance for clinical practice. The calculation is estimated on AAQ-II pre- and post-intervention change between the PIPCI group and the active control group Sample size was calculated with the R package SIMR, which allows power calculations of Linear Mixed Models (LMM) and is based on Monte Carlo simulations (Green and Macleod, 2016). The sample size estimate is based on changes in our primary outcome, AAQ-II. The model included the interaction of time (pre- versus post-test 1; continuous) and group (experimental versus active control; factor). The model also had a subject ID (factor) as a random effect to account for repeated measurements. We used the power curve function to explore trade-offs between power and sample size. The analysis showed that to get a significant difference between the experimental group and the active control group we need at least 35 participants (to have 93% power) in each group. We expect a 15% attrition rate (Norton et al., 2014), so initial recruitment of 120 (40 in each group) participants should ensure that about 105 participants remain in the final sample. The output of the power analysis is shown in Figure 3.

### Ethical Considerations

The project has been ethically approved by the Regional Ethical Committee in Stockholm (Dnr. 2018/2057-31) and follows the Declaration of Helsinki. The included patients will provide signed written consent. The collected data will be handled according to the general data protection regulation (GDPR), and according to applicable legislation to ensure an optimal protection of patients’ security. Pseudonymization will be used. Project-specific identifiers will be assigned to all participants and those IDs are to be used throughout the study. A lookup key file that links the Project ID to the participant’s personal identity number will be kept in a secure server at Karolinska Institutet, Division of Clinical Geriatrics, Department of Neurobiology, Care Sciences, and Society were the chief investigator works together with the other main researchers.

Cognitive impairments are often related to a progressive decline in cognitive and intellectual abilities. There is therefore a risk that patients may progress in their decline during the period in which they partake in the present study. Clinicians in the project will need to be watchful on potential progression and initiate a referral to other health care units that may be needed if the patient’s condition worsens. The intervention might in some cases be perceived as psychologically and emotionally challenging, for instance, the behavioral change part. This is not unique for the current study but is often a part of any psychological clinical treatment. In addition, there may be patients that experience frustration or are disappointed by a (lack of) improvement, in relation to their expected gains following treatment. These potential negative side effects of participation will be systematically monitored and addressed professionally. To provide quality-assured support to the patients, clinicians (licensed or under supervision to be licensed), psychology students under clinical training or psychology graduated (MSc) assistants will interact with the participants. Most of them will be licensed and sanctioned by the Social Board of Health and Welfare (Socialstyrelsen) and therefore expected to follow the ethical guidelines to protect the patients and their personal information.

The targeted patient group is seldom offered any psychological treatment or support as part of their clinical routine. Therefore, from an empirical perspective, we cannot identify any known disadvantages of participation in the present study. Rather, the present study provides a new venue of treatment/rehabilitation methods that has been completely lacking for these patients. Potential benefits should therefore outweigh the potential risks for the individual when partaking in the present study.

Thus, we consider that the present design has minimal risk for these participants.

### Amendments

We have not yet decided the method for analyzing the measures derived from blood and saliva, this will be decided in collaboration with experts in the field and researchers from outside the present research group. As soon as the analytical strategy for evaluating the biological measures are decided, we will update this information on ClinicalTrials.gov. In case of additional amendments to the present study protocol, which might impact patient safety, ethical aspects, or scientific evaluation of the trial, we will submit a protocol amendment to the Regional Ethical Committee in Stockholm and await their approval. Furthermore, our trial registry in ClinicalTrials.gov will be updated.

### Adverse Events

An adverse event is generally defined as an event that occurs during the research project that results in a worsening of symptoms for the participants, i.e., that involves somatic or psychological harm. Clinical (psychologist and MD) judgment will decide the level of seriousness of a potential adverse event according to standard clinical practice. In addition, a deterioration in the outcome measures can be defined as indicative of adverse events. All adverse events will be reported. In case of a more serious adverse event, we will consult our psychiatric expert, and referral to specialist health care will be conducted as deemed appropriate.

### Insurance

Insurance coverage will be provided by Landstingets Ömsesidiga Fond (LÖF) that is a Swedish insurance company whose main task is to insure publicly financed health care providers. If a patient suffers an injury while in the research study, the injury will be evaluated by LÖF and may result in financial compensation according to the Patient Injury Act.

### Statistical Analysis

#### Strategy for the Main and Secondary Hypotheses Analyses

The continuous longitudinal data derived from the RCT will be analyzed using appropriate growth models (e.g., LMM) that in addition to studying change at the group level also can: model change on the individual level; flexibly incorporate time-varying predictors; handle dependency for repeated observations and provide correct estimates with missing data under largely unconstrained missing data conditions (Hesser, 2015). As a default strategy we will use LMM to analyze the condition by time interaction and will follow established recommendations for model specification and reporting results (Bolger and Laurenceau, 2013). In the main analysis, we will compare intervention change between the PIPCI group and the active control group. However, we will also compare the PIPCI group and the active control group with the waiting list group. An alpha level of 0.05 and a 95% CI will be used to evaluate the main and secondary analyses. Regarding the LMM-analyses, assumptions relating to the normal distribution of residuals and homogeneity of variance will be assessed, respectively, based on visual evaluation of a histogram of model residuals, as well as by a plot of the model fitted values against the residuals from the model.

#### Strategy for the Exploratory Research Questions

##### Question 1

In addition to using the growth model framework to investigate predictors of intervention outcomes, we will also apply non-linear models using machine learning methodology, such as support vector machines (SVMs). The SVM fits the separating hyperplane with support from the cases that lie closest to each other in hyperspace but which are of different labels, the support vectors. The SVM is therefore less sensitive to outliers than the classic linear model. Moreover, by transforming the hyperplane with a non-linear kernel, the SVM can also separate (classify) non-linearly separable classes. The developed risk models will mainly predict cognitive test measures (neuropsychological assessments), and biological markers (visual ratings derived from magnetic resonance imaging, and cerebrospinal fluid dementia measures.

##### Question 2

The qualitative approach will be conducted in line with consolidated criteria for reporting qualitative research (COREQ) (Hayes et al., 2006), with the aim to examine in-depth participant’s experiences of participation in the RCT. Interviews will be conducted with participants in the study after the long-term follow-up. Interviews will be digitally recorded and transcribed, and content analysis will be used in the analysis (Graneheim et al., 2017).

##### Question 3

The QALY calculation will be evaluated by the change in AAQ-II induced by the treatment multiplied by the duration of the treatment effect to provide the number of QALYs gained (Prieto and Sacristán, 2003). QALYs will then be incorporated with medical costs to generate a final common denominator of cost.

#### Sensitivity Analyses

Importantly, in line with the intention-to-treat principle, all available randomized participant data will be analyzed using maximum likelihood estimation, an approach that uses information from all available observations to estimate parameters and provides unbiased estimates and standard errors in the presence of incomplete data under the assumption that data are missing at random. Appropriate sensitivity analyses will be performed to assess the robustness of the findings, and to identify possible key limitations, e.g., the effects of dropout.

### Dissemination Policy

We will communicate our research findings via several channels:

• Research articles in high impact scholarly journals
• National and international research collaborations
• Presentations at local unit meetings, and hospital committee meetings
• Presentations at national or international conferences.
• Presentations at patient organizations
• Communication to patients and their close ones at the clinical settings
• Via media communication, such as press releases
• Validation studies of our research findings at other research units/hospitals.

## Discussion

The main purpose of this article was to introduce the novel psychological intervention (PIPCI) manual and to report the study protocol for evaluating the intervention. We consider the present study methodology adequate to generate reliable and valid results on feasibility, intervention effects, and predictions of outcome based on symptomology and biomarkers.

Cognitive medicine is a new but emerging field intersecting the health-care sciences and neuroscience. Cognitive medicine aims to uncover diverse cognition-related disease mechanisms, with an overarching focus on how to cope with or to prevent cognitive decline (Wallin et al., 2018). The development and evaluation of a feasible and effective psychological intervention manual has the potential to provide a new intervention as well as important information regarding non-pharmacological treatments for a common and many times debilitating somatic condition that affect the afflicted individual, significant others, the local community and the society at large. If the psychological intervention shows positive effects on life satisfaction, psychological health, biological changes, and healthcare costs, it will have important implications on the future healthcare of patients with early CIs that are at risk of developing dementia.

To prevent falsely positive trial effects, an active control group will be recruited. It is important to include a placebo-like condition so that the experimental group does not have a better prognosis than the comparison group for reasons other than the hypothesized effects of the intervention during the study (Van De Ven et al., 2016). The active control intervention is related to cognitive training and we cannot neglect the possibility that the active control group improves similarly to, or even better (especially on the cognitive measures) than the experimental group which will receive PIPCI. Thus, a strength of our study design is the control condition, which increases the possibility to draw causal conclusions of not only intervention effect but also intervention specificity.

In a later phase, the evaluated psychological intervention has the potential to be disseminated and scaled to group-, and/or internet-based administration, as well as delivered in hybrid modalities including both face-to-face (in individual and/or group settings) and internet-based elements. This could make the intervention more available for individuals that cannot easily visit adequate healthcare facilities and could potentially also increase the cost-effectiveness of the intervention. We will not consider cognitive MCI-subtypes at the inclusion occasion. However, we will evaluate if cognitive MCI-subtypes predict intervention outcomes. Cognitive subtype in this regard refers to the pattern of impairments across the cognitive domains and is not a specific diagnosis. SCD or MCI can be due to any underlying condition. Finally, a future prospect is also to evaluate the psychological intervention for those that struggle with adherence to the FINGER intervention to increase motivation to perform lifestyle changes.

## Conclusion

The new psychological intervention (PIPCI) manual was described, and details were reported on the clinical trial that will evaluate its efficacy and cost-effectiveness. We expect that the project will provide improved support for those who suffer from psychological symptoms in relation to their early phase CIs. In addition, we expect the method to be useful for those who struggle with adherence to lifestyle changes (i.e., increase patient motivation to adhere to the FINGER intervention). The underlying causes of CIs differ due to a heterogeneous etiology, but the implications are many times similar, and if patients learn efficient strategies and approaches to handle these implications, their functioning in daily living and psychological responses to CI will likely benefit. Therefore, if significant intervention effects are seen, it may be possible to implement the intervention also for individuals with other brain conditions, such as traumatic brain injuries or stroke. The underlying causes of CIs often differ due to heterogeneous etiology, but the implications are many times similar. And patients that learn efficient strategies and approaches to handle challenges in their daily living, as well as a negative emotional and psychological response to CI, will eventually benefit. However, this requires follow-up trialing.

## Ethics Statement

The studies involving human participants were reviewed and approved by the Regional Ethical Committee in Stockholm (Dnr. 2018/2057-31). The patients/participants provided their written informed consent to participate in this study.

## Author Contributions

UE led the development of the psychological intervention manual, with contributions of MKK, RW, AR, and JW. UE, JW, MKK, LH, AR, UA, and MK contributed to the study design. EW contributed with expertise in statistics and MRI. TN contributed with expertise in prevention interventions. All authors contributed to the interpretation of the findings. UE wrote the first draft of the manuscript, all authors revised it critically and approved the final version.

## Conflict of Interest

The authors declare that the research was conducted in the absence of any commercial or financial relationships that could be construed as a potential conflict of interest.

## Footnotes

1 https://karolinskatrialalliance.se/en/

## Supplementary Material

Supplementary Figure 1 Illustrating the development and evaluation process. The figure is inspired by Craig et al. (2008), BMJ Publishing Group.
