# Supplementary material for: Evaluation of a Novel Psychological Intervention Tailored for Patients With Early Cognitive Impairment (PIPCI): Study Protocol of a Randomized Controlled Trial
Source: Front Psychol. 2020 Dec 23;11:600841. doi: 10.3389/fpsyg.2020.600841 (PMC7785936; doi:10.3389/fpsyg.2020.600841)
Supplement: Supplementary Figure 1 — Illustrating the development and evaluation process. The figure is inspired by Craig et al. (2008), BMJ Publishing Group. [file Image_1.pdf]

Development of the psychological intervention manual

The research project consists of two phases, protocol development and intervention evaluation, i.e., a sequential process where the intervention manual is developed in an iterative process and subsequently evaluated. Below follows a brief description of the phases.

The development phase consists of:

1. Systematic literature search.
2. Semi-structured interviews (90 minutes) with 2 individuals with SCD, and 2 individuals with MCI, as well as several discussions with clinicians. With the literature search and interviews as a background, we generated **version 1** of the intervention manual.
3. Expert consultation. **Version 1** of the intervention manual was revised by a multidisciplinary team of experts in cognitive impairments and/or CBT/ACT. According to their comments, **version 2** was then generated.
4. Expert consultation. Version 2 of the intervention manual was revised by multidisciplinary team of experts in cognitive impairments and/or CBT/ACT. According to their comments, **version 3** was generated.
5. Pilot-studies, single-subject design.(26) 8 cases will be treated with **version 3**. The generated information from the pilot-studies will be used to create **version 4** of the intervention manual. This version will then be modified once again via the expert consultation group described above before **version 5** of the intervention manual will be generated and then used for the RCT intervention phase.

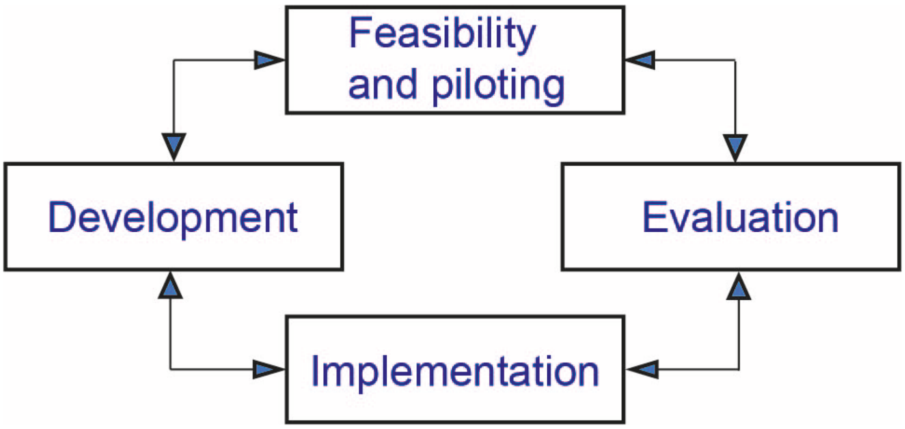

Figure 1. The psychological intervention manual was developed with a developmental mixed method according to the Medical Research Council guidance.
